# Properties of Degradable Polyhydroxyalkanoates Synthesized from New Waste Fish Oils (WFOs)

**DOI:** 10.3390/ijms241914919

**Published:** 2023-10-05

**Authors:** Natalia O. Zhila, Evgeniy G. Kiselev, Vladimir V. Volkov, Olga Ya. Mezenova, Kristina Yu. Sapozhnikova, Ekaterina I. Shishatskaya, Tatiana G. Volova

**Affiliations:** 1 Institute of Biophysics SB RAS, Federal Research Center “Krasnoyarsk Science Center SB RAS”, 50/50 Akademgorodok, Krasnoyarsk 660036, Russia; evgeniygek@gmail.com (E.G.K.); kristina.sap@list.ru (K.Y.S.); shishatskaya@inbox.ru (E.I.S.); volova45@mail.ru (T.G.V.); 2 Basic Department of Biotechnology, School of Fundamental Biology and Biotechnology, Siberian Federal University, 79 Svobodnyi Av., Krasnoyarsk 660041, Russia; 3 Centre for Advanced Protein Use Technologies, Kaliningrad State Technical University, Sovetsky Avenue, 1, Kaliningrad 236022, Russia; vladimir.volkov@klgtu.ru (V.V.V.); mezenova@klgtu.ru (O.Y.M.)

**Keywords:** degradable polyhydroxyalkanoates, PHA, biosynthesis, waste-fish oils, fatty-acid content, composition, properties

## Abstract

The synthesis of PHA was first investigated using WFOs obtained from smoked-sprat heads, substandard fresh sprats, and fresh mackerel heads and backbones. All the WFOs ensured the growth of the wild-type strain *Cupriavidus necator* B-10646 and the synthesis of PHA, regardless of the degree of lipid saturation (from 0.52 to 0.65) and the set and ratio of fatty acids (FA), which was represented by acids with chain lengths from C14 to C24. The bacterial biomass concentration and PHA synthesis were comparable (4.1–4.6 g/L and about 70%) when using WFO obtained from smoked-sprat heads and fresh mackerel, and it was twice as high as the bacterial biomass concentration from the fresh sprat waste. This depended on the type of WFO, the bacteria synthesized P(3HB) homopolymer or P(3HB-*co*-3HV-*co*-3HHx) copolymer, which had a lower degree of crystallinity (C_x_ 71%) and a lower molecular weight (M_n_ 134 kDa) compared to the P(3HB) (M_n_ 175–209 kDa and C_x_ 74–78%) at comparable temperatures (T_melt_ and T_degr_ of 158–168 °C and 261–284 °C, respectively). The new types of WFO, studied for the first time, are suitable as a carbon substrates for PHA synthesis. The WFOs obtained in the production of canned Baltic sprat and Baltic mackerel can be considered a promising and renewable substrate for PHA biosynthesis.

## 1. Introduction

Degradable polyhydroxyalkanoates (PHAs—polyesters of microbiological origin) are currently considered promising candidates for the gradual replacement of synthetic non-destructible plastics. The world production of synthetic plastics has increased to 400 million tons per year; it is expected that by 2050, it will reach 1.0 Gt per year [1]. The accumulation of plastic waste obtained from non-renewable resources creates a global environmental problem [2,3]. The solution to the problem of plastic waste is associated with the need to recycle it, despite the high associated costs [4]. According to the United Nations Environment Program (UNEP) [5], the organization and implementation of the plastic cycle on a global scale will significantly reduce the pollution of the biosphere with plastic waste and will help to reduce the greenhouse footprint and greenhouse-gas emissions in the global plastic life cycle. The second way to solve this problem is associated with a gradual transition to a new generation of destructible polymer materials [6], among which polyhydroxyalkanoates occupy a special place.

The PHA is a class of biodegradable thermoplastic polymers with a wide range of chemical structures and physicochemical properties [7,8,9,10,11,12,13,14]. These can be processed into products using available methods from various phase states [15,16], as well as to obtain composites with various fillers [17]. These useful properties, combined with destructibility and high biocompatibility, make PHA one of the most promising materials for use in various areas, including agriculture and biomedicine [18,19,20].

The key problem for increasing production volumes and expanding the scope of PHAs is to reduce their cost by using available carbon raw materials. Potentially, various substrates can serve as raw materials for PHA synthesis, as individual compounds [19,20,21,22,23,24,25,26,27] and, of particular significance, various byproducts (products of the processing and hydrolysis of plant raw materials, byproducts from different industries [27,28,29,30,31]. The potential for PHA synthesis in byproducts of various origins has significant potential for “The Circular Economy” [14,32,33,34]. This is aimed at reducing the accumulation of waste in the biosphere, as well as at increasing the efficiency of industrial production, since the complex processing of raw materials increases the efficiency of their use. This is due to the fact that this creates the possibility of collecting the waste generated through their subsequent processing for an intended purpose, depending on the origin and chemical composition.

Therefore, in the food industry, a significant problem is the lack of a rational technology for processing large amounts of fat-containing waste. The authors of [35] calculated that the amount of waste fat generated annually around the globe is about 29 million tons; this includes fatty acids, low-grade oils of vegetable and animal origin, and the waste generated during their processing. Used cooking oils are now applied in the production of biodiesel. The synthesis of various target products, including biodegradable plastics, particularly PHA, can become a new direction for the use of such large-tonnage fatty byproducts.

The interest in lipid substrates for PHA synthesis emerged relatively recently. Readers should note a review by colleagues from Malaysia [36], which considers the results obtained and published to date and discusses the prospects for the synthesis of PHA on vegetable fat-containing substrates of various origins, including vegetable oil from various sources (palm oil, soybeans, olives, peanuts, jatropha oils, canola, rubber-seed oil, and date-seed oil). The review’s authors analyzed publications from the period of 2010–2020, which showed the possibility of PHA synthesis on vegetable oils of various origins. 

In another review paper [37] the mechanisms of PHA synthesis at the molecular level and the prospects for the use of fat-containing substrates for PHA synthesis by producers of various taxa were also discussed, including *Pseudomonas mosselii* TO7, which synthesizes MCL–PHA when grown in C8–C12 unsaturated fats, palm kernels, and soybean oils, *Delftia acidovorans* DS-17, capable of utilizing triacylglycerols, *Aeromonas caviae*, which accumulates P(3HB-*co*-3HHx) when grown in soybean oil, recombinant strains of *Ralstonia eutropha*, and *P. putida*. In addition, the authors showed the efficient use of waste vegetable oil for the synthesis of MCL–PHA by *Pseudomonas* sp. Gl01.

The processing products of low-grade animal fats are also under investigation for the synthesis of PHA. In the series of works by the Technische Universität Berlin, Institute of Biotechnology, Chair of Bioprocess Engineering, under the direction of Dr. S. Riedel, PHA products were investigated using low-quality animal by-products as C-substrates (used oil for frying from snack bars, processed animal fats from various agricultural animals (pork, poultry, pig/beef, mixed, and beef) [38,39,40]. It is important to note the energy efficiency of the transformation of lipid substrates in the processes of microbial metabolism. The theoretical yield of PHA on lipid substrates can reach 0.6–0.8 g/g, which is practically twice as high as that using sugars. With sugars, the yields are 0.28–0.37 g/g for glucose [41,42,43] and 0.12–0.35 for fructose [41,44,45,46].

The problem of the utilization of fat-containing waste is relevant to the fish-processing industry, among others. Fish waste is currently underused. The search for new technologies for the processing of such waste not only to obtain fishmeal [47,48], but also to obtain high-demand products with high added value (fish oil, omega-3 fatty acids, protein products, etc.) is very relevant. Fat-containing waste obtained in the process of fish processing is a new, but still poorly studied source of carbon raw materials, which can become effective and renewable substrates for various biotechnological processes, including the production of PHA.

The content of fat-containing waste is estimated to be up to 60% of the volume of fish by-products [49]. Globally, the byproducts from the fish-processing industry exceed 20 million tons/year, representing 25% of the total marine-fisheries catch. At the same time, about 70% of fish is processed before sale, resulting in the formation of 20–80% of solid fish waste, depending on the level of processing. These byproducts must be recycled in some way to prevent environmental pollution, or disposed of in a safe way [50].

However, the study of fat-containing fish-processing waste as a substrate for the synthesis of PHA began relatively recently. The available research so far includes a small number of works. In a series of works by colleagues from Vietnam, where a huge amount of waste is generated annually during fish processing, the results of PHA syntheses by various producers included the halophilic bacterium *Salinivibrio* sp. M318 [51], and isolates from the genus *Ralstonia* [52] and wild-type *Cupriavidus necator* H16 strain [53]. Using waste fish oils (WFOs) from various sources (a mixture of used fish oil and glycerin, crude fat and fat from Basa (*Pangasius bocourti*) fish waste), researchers obtained in flasks biomass concentrations and PHA contents of 5–10 g/L and 50–70%, respectively, and up to 50 g/L to 117 g/L and 82.9%, respectively, in a fermenter. The synthesis of PHA using hydrolyzed fat from *Alaska pollock* [54] was studied. The synthesis of P(3HB) using extracts of fatty acids taken from fish-processing waste in a culture from a *Bacillus subtilis* strain (KP172548) was described in [55]. Skipjack tuna (*Katsuwonus pelamis*) condensate from Songkla Canning Co., Ltd., Thai Union Group (Thailand), rich in organic substances and a by-product of the canning industry, was evaluated as a substrate for PHA synthesis in a culture from *Cupriavidus necator* TISTR 1095 [56]. Several studies have investigated the synthesis of PHA from fats extracted from waste effluents from the fish-canning industry [57,58].

In general, the results obtained are encouraging and show that WFO can become a large-scale and energy-efficient substrate for the production of degradable bioplastics. At the same time, the achieved production indicators in terms of the yield, composition, and properties of PHA differ significantly. This is due to the fact that bacteria from different taxa were used for the PHA syntheses, and the cultivation processes took place under different conditions and regimes, and with different carbon sources (different fish species and different methods of obtaining fat). Therefore, in each case, when working with new lipid substrates, it is necessary to select strains and appropriate conditions for efficient polymer synthesis. The attraction of a new carbon substrate makes it necessary to study the production parameters of the process, as well as the composition and properties of PHA. It should be noted that the production parameters of PHA-synthesis processes using fat-containing substrates are still lower than those using carbohydrates. This invites research aimed at finding new sources of fat-containing waste.

The purpose of this work is a comparative study of the synthesis and properties of PHA in the culture of the wild strain *Cupriavidus necator* B-10646 using unstudied waster fish oils (WFOs), differing in terms of the composition of fatty acids, obtained by processing Baltic sprat *(Sprattus sprattus balticus*) and Atlantic mackerel (*Scomber scombrus*).

## 2. Results

Three different sources of WFO, the heads of smoked Baltic sprats (*Sprattus sprattus balticus*), fresh Baltic sprats of substandard quality, and the heads and backbones of fresh Atlantic mackerel (*Scomber scombrus*), were studied, for the first time, as the sole carbon sources in the nutrient medium for PHA synthesis. The choice of these sources was due to the large volume of these WFOs. Thus, the global catch used for the production of canned sprats is about 500 thousand tons per year [59]. Canned sprats are a large-capacity product produced in many countries, including the Baltic, the Black Sea, the Caspian countries, the Faroe Islands, Norway, and Russia. About 20.0 tons of fish waste (heads, entrails, backbones, and other fish waste from cutting for food purposes) is generated per day at fish-processing enterprises in the Kaliningrad region [60]. Simultaneously, about 10.0 tons of waste per day is due to sprats, in the form of smoked sprat heads. These byproducts are concentrated in landfills as municipal solid waste and incinerated. This makes it expedient to find solutions for the use of this fat-containing waste, including as a potential raw material in the processes of obtaining target biotechnology products [61].

Atlantic mackerel are among the 23 most heavily caught species globally, with global catches of up to 2.5 million tones in some years (FAO, 2014). Waste and losses in the processing of mackerel are about 30–35%. The heads are the largest fraction formed during the processing of mackerel (at least 23.41% of the mass of the feedstock) [62].

### 2.1. Characterization of WFOs Derived from Various Byproducts

The animal and vegetable oils obtained from natural raw materials are multicomponent substances, principally consisting of triacylglycerols (TAGs), in which fatty acids (FAs) are joined to the glycerol backbone. The composition of oils and the fatty acids they contain depend on the source of production (heads, entrails, fins, backbones, etc.) and the technology used for their extraction and purification [63]. The composition of WFOs depends on the specific species of fish, their trophism, and their habitat.

The compositions of the studied WFOs revealed some differences (Table 1). The highest lipid content (99.3%) and the lowest nitrogen content (0.284% and 1.78%) were in the WFOs obtained from smoked-sprat heads. The lipid contents in the fat samples obtained from fresh sprats and fresh mackerel heads and backbones were comparable, at 86.2% and 89.6%, respectively, with higher total nitrogen contents (0.309% and 0.503%, respectively).

The metabolism of lipid substrates in bacterial cells is determined by the action and activity of lipolytic enzymes, which ensure the availability of fatty substrates for microorganisms by hydrolyzing them extracellularly [64]. In [65], during the cultivation of bacteria *Alcaligenes eutrophus* (later taxonomic names: *Ralstonia* and *Cupriavidus*) on olive oil as a carbon substrate, it was shown that the producer possesses the enzyme lipase, which is produced extracellularly. It has been suggested that lipase hydrolyzes lipid substrates, resulting in the formation of fatty acids that enter bacterial cells and are metabolized to acetyl-CoA as a result of FA *β*-oxidation reactions. In subsequent works, this assumption was confirmed, and it was shown that lipases provide the hydrolysis of triacylglycerols (TAG) into diacylglycerols (DAG), monoglycerols (MAG), glycerol, and free fatty acids (FA); as a result, a complex lipid substrate of plant or animal origin becomes available to cells, penetrates into them, and is metabolized [38,66,67,68]. Therefore, information on the composition of fatty acids in lipid raw materials is of great importance for the process of the microbiological synthesis of PHA.

The *C. necator* B-10646 strain used in this work had lipase activity, which ensured its growth and PHA synthesis on various vegetable oils: palm, sunflower, and Siberian oil-seed oils [69]. The determination of the activity of the lipolytic enzyme of *C. necator* B-10646 showed its presence in cells grown on a medium with sugars in the absence of fats (0.4 U/mL). When replacing sugars with oils obtained from all three sources, the lipase activity of the cells increased to 6.6–8.4 U/mL. This indicates the ability of the strain to metabolize fatty acids of the studied oils and the constitutive nature of the enzyme.

The fatty-acid compositions of the studied WFOs are shown in Table 2.

In the fatty-acid composition of the lipids obtained from the smoked-sprat heads, 20 FAs with chain lengths from 14 to 24 carbon atoms were identified. Among the dominant FAs, palmitic (28.04%), oleic (25.33%), docosahexaenoic (16.73%), and timnodonic (8.74%) acids were identified. The contents of other fatty acids with a C-chain length of 18 carbon atoms (linolenic, stearic, linoleic, and myristic), were much lower (3.0–4.5%). A low content (1.1–1.5%) of long-chain (C20 and C24) monoenoic fatty acids was noted. The minor FAs (with contents of less than 1.0%) included saturated fatty acids with C-chain lengths of 15, 20 m and 22 carbon atoms, as well as monoenoic fatty acids C16:1 and C17:1. In small quantities, iso- and anti-iso fatty acids from the C14–C16 series were also found. The total content of saturated fatty acids was 38.15%; the monoenoic value was 28.67%, while polyenoic fatty acids accounted for 32.78%. The saturation factor was 0.62.

The 33 fatty acids were identified in WFOs from the fat waste of the fresh sprats. Palmitic (18.80%), oleic (17.74%), and docosahexaenoic (22.28%) acids were identified as the dominant acids. The FA composition contained various branched fatty acids with a chain lengths of 14 to 22 carbon atoms and polyene C20–C24 FAs, which are absent from the fat waste of sprat heads after smoking. The total contents of saturated, monoenoic, and polyenoic fatty acids were 34.2%, 29.19%, and 36.65% of the total FA, respectively. The saturation factor was 0.52.

The WFOs obtained from the heads and spines of the fresh mackerel contained 29 FAs. Palmitic (26.21%), oleic (32.70%), and docosahexaenoic (16.94%) acids were also identified as dominant. In the composition of the FAs, a lower content of polyene FAs (24.04%) was noted, in contrast to the fat waste from the fresh sprats (36.65%) and smoked-sprat heads (32.78%). In addition, the composition of FAs did not contain polyene FAs C20–C24, identified in the fat waste from the fresh sprats. The total contents of saturated and monoenoic fatty acids were 39.32% and 36.66%, respectively; the saturation factor was 0.65.

The published data on the composition of FAs in WFO lipids, which are studied as substrates for PHA synthesis, are scarce. In [54], crude fat hydrolysates from pollock byproducts were characterized (with a saturation coefficient of 0.35); it was shown that they were represented by fatty acids with C-chain lengths of 14–22 carbon atoms, with monoenoic fatty acids (28.0%) as the predominant type, as well as acids with a C-chain length of 20 carbon atoms. Another type of WFO obtained at the fish market (Bhubaneswar, Odisha (India)) from solid fish waste, including fish intestines and scales, was characterized by a content of 58.0% protein and 19.0% minerals and lipids; the contents of palmitic and oleic fatty acids were estimated to be up to 22.0%. The composition of the spent fat from bass fish (with a saturation coefficient of 0.84) was dominated by unsaturated fatty acids (more than 55%), as follows: oleic acid (up to 38.6%), palmitic fatty acid (up to 30.6%), linoleic, stearic, myristic, and palmitoleic fatty acids (up to 9.0, 8.2, 4.2 and 2.5%, respectively) [51,52,53].

A comparison of the WFOs studied in this work with previously published data on WFOs showed that sprat oil is similar in FA composition to pollock fat, but contains more docosahexaenoic and palmitic acids; it also contains linolenic fatty acid, while eicosatetraenoic fatty acid is absent. When comparing the compositions of basa-fish waste and sprat oil, it was shown that the differences are more significant: unlike sprat oil, basa-fish waste is characterized by a low content of polyunsaturated long-chain fatty acids. The difference in the composition of lipid FAs obtained from fresh sprats is in the presence of various branched FAs with carbon-chain length from 14 to 22, as well as the presence of long-chain polyenoic acids. In the fat waste from the heads and backbones of the fresh mackerel, a low content of polyenoic fatty acids was noted.

### 2.2. Cupriavidus Necator B-10646 Growth and Polyhydroxyalkanoates Synthesis on Different WFOs

**Figure 1:**
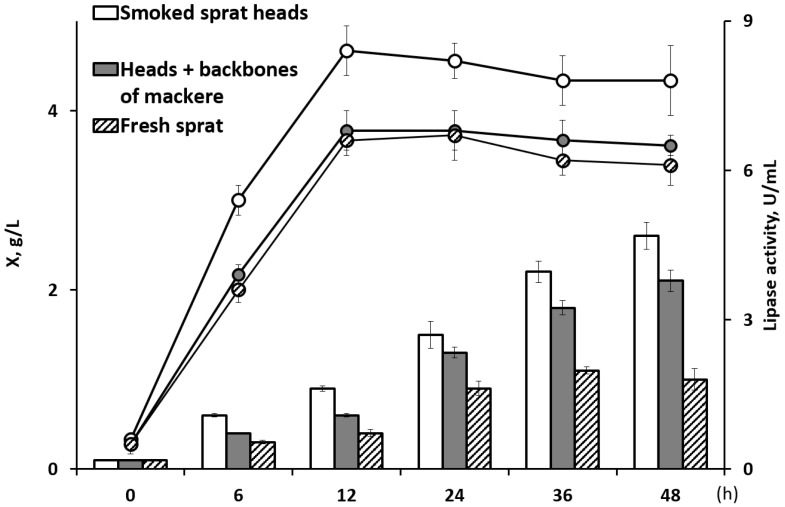
Biomass concentration of *C. necator* B-10646 bacteria (bars—X, g/L) and lipase activity (points—U/mL) in various sources of WFO.

The results of a preliminary assessment of the ability of the *C. necator* B-10646 bacteria to grow on the studied WFOs are shown in Figure 1. The concentration of oils in the composition of the nutrient medium in all the variants was 10.0 g/L, the nitrogen source (NH_4_Cl) was 1.0 g/L, and the duration of the cultivation of the bacteria in batch culture was 48 h. All three sources of WFO proved to be suitable for growing this bacterial strain. The highest bacterial biomass concentration (about 2.6 g/L) was obtained when the bacteria were grown in the oil extracted from the smoked-sprat heads. The indicator was somewhat lower (2.1 g/L) in the case of the bacterial growth in the oil from the heads and backbones of the fresh mackerel. The lowest bacterial biomass yield (1.6 g/L) was obtained when using fresh sprat oil.

The data on the bacterial growth were consistent with the lipase activity, which reached a maximum at 12 h of cultivation and was measured at 8.4, 6.8, and 6.6 U/mL in the WFOs from the smoked-sprat heads, the heads and backbones of the fresh mackerel, and the fresh sprats, respectively. 

**Figure 2:**
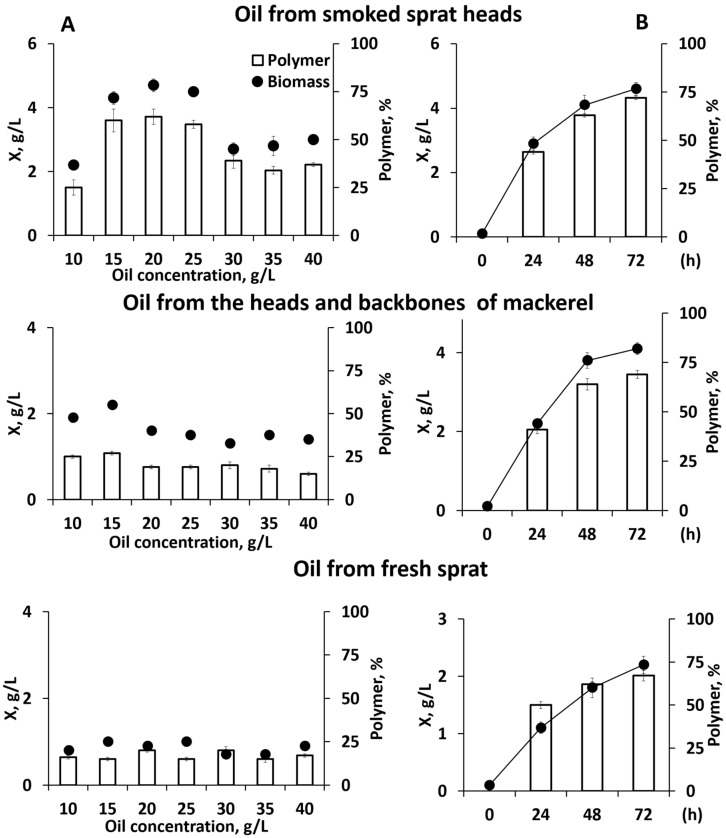
Indicators of *C. necator* B-10646 grown on WFO: (**A**)—influence of the type and concentration of the WFO on the bacterial biomass (X, g/L) and the intracellular concentration of PHA (%); (**B**)—bacterial biomass concentration and PHA content in the PHA-synthesis mode with nitrogen-growth limitation.

Since the substrate components have physiological limits of action specific to various producers, the growth and synthesis of PHA by the *C. necator* B-10646 bacteria was studied at various concentrations of the WFO in the medium. The bacterial biomass production and the PHA synthesis by bacteria were studied on a complete nutrient medium (NH_4_Cl concentration 1.0 g/L), in the absence of conditions limiting the growth of the producer, while varying the concentration of the WFO in the medium from 10.0 to 40.0 g/L (Figure 2A).

The highest indicators of the bacterial biomass concentration (4.3–4.7 g/L) when the *C. necator* B-10646 was grown in the oil from the sprat heads were obtained in the concentration range from 15.0 to 25.0 g/L. A higher concentration (30.0–40.0 g/L) inhibited the growth of the bacteria and caused a decrease in the bacterial biomass. A decrease in the concentration of this WFO to 10.0 g/L limited the growth of the bacteria. The maximum polymer content in the cells (60–62%) of the weight of the absolutely dry biomass was obtained at oil concentrations of 15.0–25.0 g/L. At lower and higher oil concentrations, the polymer content did not exceed 40%.

When using oil obtained from the heads and backbones of the fresh mackerel as the sole source of carbon, the values were lower. The maximum values of X and PHA did not exceed 1.9–2.2 g/L and 25–27%, respectively, and were obtained at the lowest substrate concentrations (10.0 and 15.0 g/L). An increase in concentration to 20.0–40.0 g/L led to a decrease in the indicator to 1.3–1.6 g/L. A similar effect of the WFO concentration was also shown for the accumulation of the polymer in the cells. An increase in the concentration of this source in the medium was accompanied by a decrease in the content of PHA in cells to 15–20%. When the *C. necator* B-10646 was cultivated in WFO obtained from the fresh sprats, regardless of the substrate concentration in the medium, the bacterial growth was extremely poor. The bacterial biomass concentration did not exceed 1.0 g/L by the end of the cultivation, which lasted 48 h. The content of the polymer in the cells was also low and amounted to 15–20% (Figure 2A).

The revealed limits of the physiological action of the concentrations of the studied WFO types, which affected the growth of bacteria and the production of PHA, made it possible to further implement the process of growing the *C. necator* B-10646 bacteria in a two-stage mode, maximizing the accumulation of reserve polymers. The concentration of NH_4_Cl at the first stage (30–35 h) was reduced to 0.5 g/L (a factor stimulating the accumulation of PHA in this bacterial strain). The second stage of a similar duration was realized in the absence of nitrogen in the medium. The total fermentation time was 72 h. The concentration of WFO in the medium obtained from the heads of the smoked sprats and fresh sprats was 20.0 g/L; the concentration obtained from the heads and backbones of the fresh mackerel 15.0 g/L. The results obtained are illustrated in Figure 2B.

The maximum specific growth rates were recorded in the first 24 h. These were 0.14 h^−1^ for the WFO obtained from the smoked-sprat heads, 0.10 h^−1^ for the WFO obtained from the fresh sprat heads, and 0.13 h^−1^ for the WFO from the fresh mackerel heads and backbones (Table 3). Further, in all the variants, the specific growth rate decreased as the nitrogen was exhausted in the medium, reaching 0.01 h^−1^ by the end of the process. The intracellular concentration of PHA by the end of the first stage was comparable in all the variants, and it was about 40–50%. The maximum PHA-specific-synthesis rate also decreased during the first 24 h, and it was 0.19 h^−1^ for the WFO obtained from the smoked-sprat heads, 0.15 h^−1^ for the WFO obtained from the fresh sprats, 0.17 h^−1^ for WFO from the heads and backbones of the fresh mackerel. At the second stage of the process, the PHA-specific-synthesis rate decreased to an average of 0.04 h^−1^, and an increase in the index X, g/L (biomass concentration) reflected not the true increase in cell biomass as a result of their replication, but the accumulation of polymer in the cells formed from the metabolites synthesized during the initial stages of the process.

The highest process performance was obtained using the WFO obtained from the smoked-sprat heads (4.6 g/L) as a C-substrate; the biomass productivity was 0.064 g/L·h, and the PHA productivity was 0.046 g/L·h. This was close to the bacterial growth on the WFO obtained from fresh mackerels’ heads and backbones (4.1 g/L), with biomass and PHA productivities of 0.057 and 0.039 g/L·h, respectively. The lowest biomass concentration was obtained from the fat waste from the fresh-sprat processing (2.2 g/L); the biomass productivity was 0.031 g/L·h and the PHA productivity was 0.02 g/L·h. The polymer concentrations were similar and amounted to 67–72% for all the variants by the end of cultivation (72 h). In terms of the cell-biomass concentrations and polymer contents, the results are comparable to a similar process in the culture of this strain grown in flasks on glycerol (7.2 g/L and 71.0% PHA, respectively) [70], but inferior to the indicators obtained when using sugars (8.9–9.0 g/L and 80.0–85.0% PHA, respectively) [71].

The results obtained in this work are consistent with the available research data on the synthesis of PHA in flasks using other types of WFO (Table 4). 

It has been shown that during the cultivation of various strains of *Pseudomonas* on hydrolyzed pollock-fat waste, the biomass concentration of the producers was 1.7–4.7 g/L, with an intracellular polymer content of 6–53% [54]. The *Bacillus subtilis* bacteria (KP172548), when grown on an extract of fatty acids from WFO, synthesized PHA up to 1.62 g/L, at a cell concentration in the culture of 2.3 g/L [55]. When activated sludge from treatment facilities in fish-canning plants was used, PHA contents of 22–37% were obtained [57,58]. When tuna-processing-waste condensate was used as a C-substrate [56], the *Cupriavidus necator* TISTR 1095 strain synthesized up to 3.8 g/L of the P(3HB-*co*-3HV) copolymer at a biomass concentration of 7.5 g/L. A bacterial biomass concentration of 3.93 g/L from the *Ralstonia* M91 strain was obtained in flasks at a polymer concentration of 2.43 g/L; scaling the process in a fermenter with a volume of 10 L made it possible to increase the accumulation of the biomass and the polymer concentration to 5.32 g/L and to 2.73 g/L, respectively [52].

### 2.3. Chemical Composition and Properties of PHA Synthesized on Various Types of WFO

**Figure 3:**
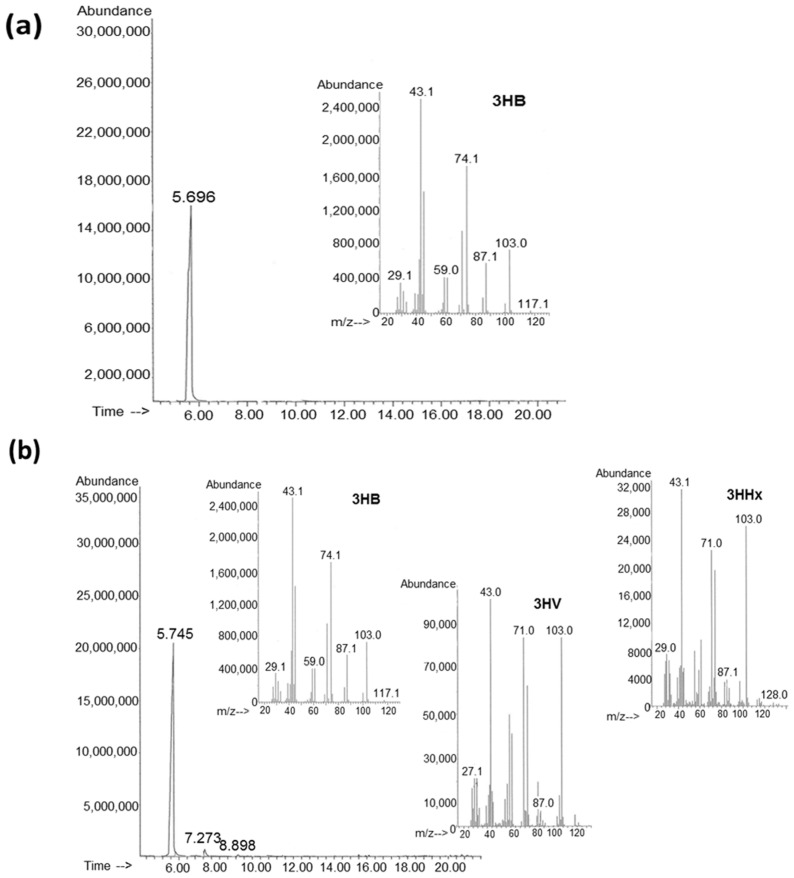
Chromatograms and mass spectra of P(3HB) (**a**) and P(3HB-*co*-3HV-*co*-3HHx) copolymer (**b**) synthesized by *C. necator* B-10646 on various WFO sources.

Differences between the studied WFO sources and their fatty-acid compositions influenced the chemical compositions of the PHAs obtained. Using the *C. necator* B-10646 bacteria during the growth on WFO from substandard fresh sprats, as well as the heads and backbones of fresh mackerel, homopolymer P(3HB) was synthesized; from the smoked sprat heads, we synthesized a three-component copolymer P(3HB-*co*-3HV-*co*-3HHx), in which 3-hydroxybutyrate (3HB) monomers (97.1 mol%) were dominant and 3-hydroxyvalerate (3HV) monomers were present (1.6 mol%), and minor inclusions of medium-chain 3-hydroxyhexanoate (3HHx) (0.3 mol.%) (Figure 3).

**Figure 4:**
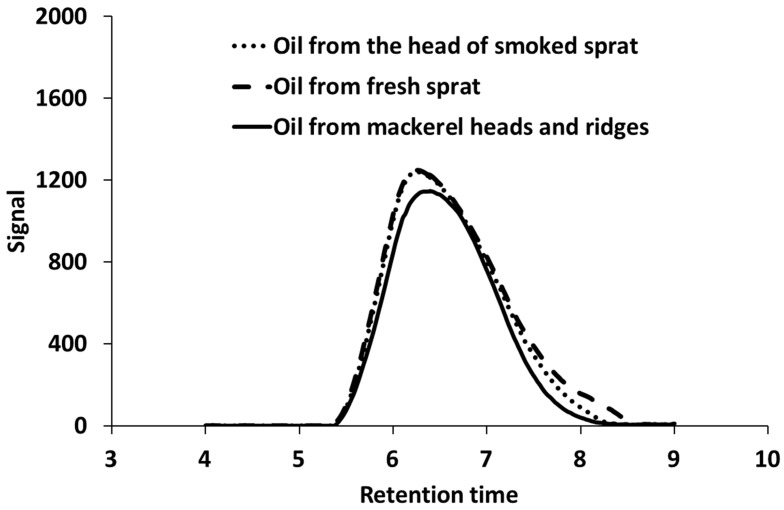
Molecular-weight-distribution chromatogram of PHAs synthesized by *Cupriavidus necator* B-10646 from various WFOs.

The chromatograms illustrating the molecular-mass characteristics of the PHA samples (Figure 4) indicate that the values of the average number (M_n_) and average molecular weight (M_w_) of the copolymer were slightly lower (752 and 134 kDa), and that the polydispersity (Đ) was higher (5.6), compared with the samples of P(3HB), in which M_n_ was 175–209 kDa, and the M_w_ was 650–711 kDa at similar values of Đ (3.4 and 3.7) (Table 5). 

An X-ray analysis showed that the degrees of crystallinity (C_x_) in the P(3HB) synthesized on the fat waste from the fresh sprats, as well as the heads and backbones of the mackerel, were 78% and 74%, respectively, which corresponds to the crystallinity for the P(3HB) synthesized on the sugars. A slight decrease in the degree of crystallinity, to 71%, was noted for the P(3HB-*co*-3HV-*co*-3HHx) copolymer (Table 5).

**Figure 5:**
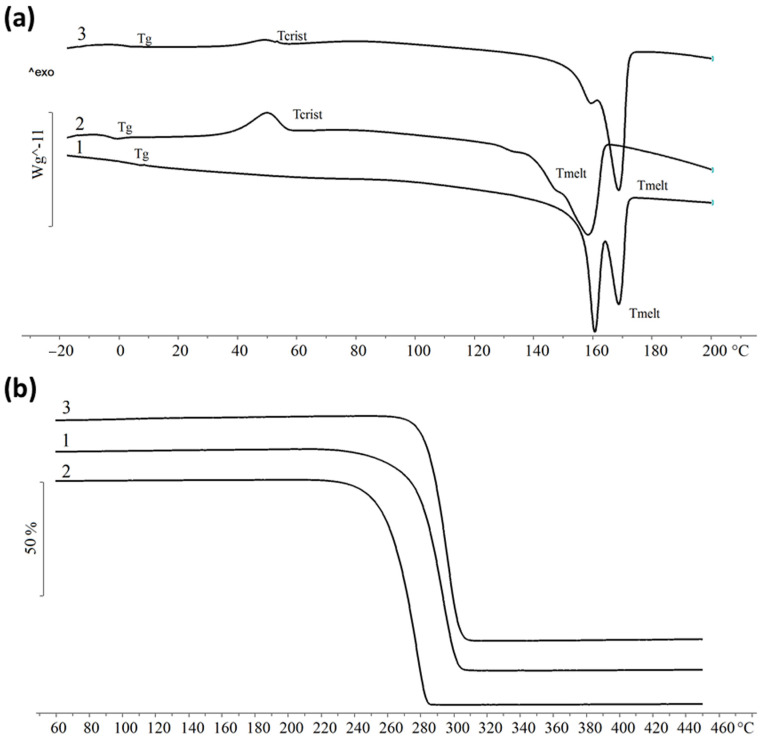
DSC curves (**a**) and thermal stability (TGA) (**b**) of P(3HB) and P(3HB-*co*-3HV-*co*-3HHx) copolymer synthesized by *C. necator* B-10646 on various WFO sources: 1—fat from smoked sprat heads; 2—fat from heads and backbones of fresh mackerel; 3—fresh sprat fat.

The important characteristics of PHA, which determine the thermomechanical properties, which in turn make it possible to process the polymers into products from melts, are their temperature properties and crystallization degrees in the native state. The presence of a gap between such thermal parameters as T_melt_ and T_degr_ determines the technological properties of the PHA obtained, since it makes it possible to obtain products during the processing of polymer melts. The thermograms obtained with the DTA and DSC methods (Figure 5) showed that the melting points (T_melt_) and degradation points (T_degr_) of the P(3HB) samples were similar, with a gap between these indicators of about 100 °C. 

All the samples had reduced melting points compared to the polymers synthesized on the sugar substrates. The lowest melting point, of 158 °C, was recorded for a sample obtained on the WFO from the mackerel heads and backbones. On the thermograms of the samples obtained from the WFO from the heads of the smoked sprats and waste from the processing of the fresh sprats, when heated, there were no large peaks of recrystallization at 49 °C, which is not typical for a homopolymer and may indicate the formation of a copolymer. The cold crystallization of these samples occurred at temperatures of 61–64 °C. The glass transition was fixed between −0.1 and −0.14 °C.

For the P(3HB-*co*-3HV-*co*-3HHx) copolymer, the presence of two peaks in the melting region at temperatures of 160 °C and 168 °C was revealed. This indicates the inhomogeneity of the sample and the formation of two crystalline phases. The crystallization of this sample occurred when cooled at 90 °C. In addition, an increase in the thermal stability of this sample was noted, in which the degradation temperature slightly increased (to 284 °C), increasing the gap to the melting point.

According to the literature data on the synthesis of PHA using various byproducts obtained in the process of fish processing, the chemical composition of polymers is significantly affected by the species specificity of the producers and the sources of the raw materials. It was shown that, in addition to the P(3HB) homopolymer, medium-chain copolymers containing 3-hydroxyhexanoate, 3-hydroxyoctanoate, and 3-hydroxydecanoate were obtained during the cultivation of various strains of *Pseudomonas* using raw pollock oil [54]. The P(3HB) obtained in that work had average molecular weights from 206,000 to 195,000 g/mol, with polydispersity values from 2.0 to 2.2, the mcl-PHA had a M_n_ of 84,000–153,000 g/mol, and the polydispersity index was in a range from 2.08 to 2.61. With regard to the *Cupriavidus necator* TISTR 1095 bacteria, it was shown that when fatty waste from the effluents of a tuna-processing plant was used as a substrate, the bacteria synthesized P(3HB-*co*-3HV) with a monomer content of 3HB up to 20 mol.%, where the M_n_ was 2000 Da, and the polydispersity was 2.5 [56]. The use of spent-bass-fish oil (*Pangasius bocourti*) as a substrate for the *Cupriavidus necator* H16 and *Ralstonia sp.* M91 strains was shown [52,53]. The authors obtained P(3HB) with the following molecular-weight characteristics: M_w_ 670 Da, M_n_ 230 Da, Ð 2.83. In another work [51], the strain *Salinivibrio* sp. M318 (VTCC910086) also synthesized PHA when a mixture of glycerol and spent-fish oil was used. In the P(3HB-*co*-3HV) copolymers obtained, the M_n_ and M_w_ varied within the ranges of 120–320 kDa and 200–630 kDa respectively; the polydispersity ranged from 1.4 to 2.0. In addition, the authors noted the presence of two peaks in the melting region for two samples with a content of 3HV monomers of 13.0 mol.% (at 141 and 160 °C) and 17.0 mol.% (at 132 and 150 °C). Using an extract of raw-fish waste as a carbon substrate, the *B. subtilis* synthesized highly crystalline P(3HB), the thermal decomposition temperature (T_degr_) of which remained almost constant (150–466 °C) [55].

## 3. Materials and Methods

### 3.1. PHA Producer Strain and Cultivation Media

The wild-type *Cupriavidus necator* B-10646 strain, which is registered in the Russian National Collection of Industrial Microorganisms (VKPM) and is able to synthesize PHA from various carbon substrates, was used [72]. The bacteria were grown in a mineral Schlegel medium [73]. To prepare the oil medium, distilled water containing Na_2_HPO_4_•12H_2_O and KH_2_PO_4_ was combined with oil. After sterilization of medium, MgSO_4_•7H_2_O, C_6_H_5_O_7_Fe•nH_2_O, and trace elements were added from sterile stocks. The NH_4_Cl was used as a nitrogen source. Fat waste from fish processing (WFO) at a concentration of 10.0 to 40.0 g/L was used as a carbon source. 

### 3.2. Obtaining and Characterizing Fat Waste from Fish Processing (WFO)

Three types of fat waste obtained from different sources were studied: the heads of Baltic sprats (*Sprattus sprattus balticus*) obtained after smoking—waste from sprat production; heads and backbones of fresh Atlantic mackerel (*Scomber scombrus*)—waste from canning production; and heads of fresh Baltic sprats of substandard quality (wrinkled, with damaged skin and other defects) (*Sprattus sprattus balticus*). The waste was provided by the enterprises in the canned-labor industry of the State Concern, “Za Rodinu,” and JSC “RosKon,” from the Kaliningrad region (RF). Separation of fat from waste was carried out in the Food Biotechnology department of the Kaliningrad Technical University by the thermal method. To this end, the crushed waste was mixed with water in a ratio of 1:1 followed by heating to 90 °C and stirring for 15–20 min, after which the mixture was centrifuged at 3000× *g* (Megafuge 1.0R, Thermo Fisher Scientific, Waltham, MA, USA). The fat fraction from the supernatant was separated by decantation.

### 3.3. Bacteria-Cultivation Technique

The producer strain was grown in flasks with a volume of 0.5–1.0 L in a shaker–incubator (New Brunswick Scientific, Edison, NJ, USA) in periodic mode: during the first 25–35 h of growth, 0.5 g/L of a nitrogen source was added to the medium, which served as a limiting factor and stimulated the production of PHA. The source of nitrogen was exhausted in the subsequent hours of growth. The inoculum was prepared by resuspending the museum culture from an agar medium in 0.5 L flasks. The initial concentration of cells in the medium was 0.1–0.2 g/L. The initial concentration of C-substrate was 10.0–15.0 g/L at an ambient temperature of 30 °C.

During cultivation, samples for analysis were taken periodically, with an interval of 24 h. The growth of the producer was determined by the optical density of the culture at a wavelength of λ = 440 nm (photoelectrocalorimeter UNICO 2100, Dayton, NJ, USA). Bacterial biomass (X, g/L) was determined by gravimetric method after centrifugation of the washed biomass at 6000 rpm (AvantyJ-HC centrifuge, BeckmanCoulter, Indianapolis, IN, USA) and drying at 105 °C for 24 h. Lipase activity was determined according to Takaç and Marul [74].

### 3.4. Analysis of the Chemical Composition of Sprat Oil

Conventional methods were used to determine the contents of carbohydrates, lipids, and protein in the C-substrates used. The fatty-acid composition of the oils was determined (according to the recommendations of Soltani et al. [75]), and the dynamics of the utilization of fatty acids of the oils by bacteria during cultivation was also evaluated. Lipids were extracted with a mixture of chloroform-ethanol (2:1). The resulting extracts were dried on a rotary evaporator, after which methyl esters of fatty acids were obtained: 1 mL of a solution of H_2_SO_4_:methanol (1:20) was added to the extract and kept for 2 h at 80 °C. The resulting fatty-acid methyl esters were analyzed using a gas chromatograph equipped with a mass detector (7890A/5975C Agilent Technologies, Santa Clara, CA, USA) and an HP-5 capillary column (Agilent Technologies, Santa Clara, CA, USA). Helium was used as the carrier gas (flow rate 1 mL/min). The temperature regime was as follows: initial temperature was 120 °C, rising to 230 °C at a rate of 5 °C/min, followed by isothermal regime for 5 min, and subsequent temperature increase to 310 °C at a rate of 10 °C/min, followed by isothermal regime for 3 min. Injector temperature was 220 °C. 

### 3.5. PHA Analysis

To determine the content of PHA in bacterial cells, dry cell biomass was analyzed, which was subjected to the procedure of methanolysis and subsequent chromatography of the resulting methyl esters of the corresponding fatty acids (7890A/5975C Agilent Technologies, Santa Clara, CA, USA) [76]. A 7890A gas chromatograph (Agilent Technologies, Santa Clara, CA, USA) equipped with a 5975C mass detector (Agilent Technologies, Santa Clara, CA, USA) was also used to determine the compositions of the PHAs obtained. To this end, PHA samples isolated from cell biomass and purified were analyzed. The extraction procedure was carried out using dichloromethane, after which the extract was evaporated (rotary evaporator R/210V, Büchi, Flawil, Switzerland) and precipitated with ethanol. In order to obtain homogeneous PHA samples that did not contain impurities, the dissolution and precipitation steps were repeated several times, after which the resulting polymer samples were dried. 

### 3.6. PHA Properties

Molecular-weight characteristics (M_w—_molecular-weight average, M_n_—molecular-number average, Ð—polydispersity) of polymers were detected using a size-exclusion chromatograph (Agilent Technologies 1260 Infinity, Waldbronn, Germany) equipped with an Agilent PLgel Mixed-C column. A DSC-1 differential scanning calorimeter (Mettler Toledo, Schwerzenbac, Switzerland) was used to analyze the thermal properties of PHA. Using the STARe software, melting point (T_melt_) was determined from exothermal peaks in thermograms. Using a TGA2 thermal analysis system (Mettler Toledo, Schwerzenbac, Switzerland), the thermal degradation (T_degr_) was determined. The X-ray analysis of structure and crystallinity determination of the polymers obtained was carried out using a D8ADVANCE X-ray powder diffractometer (Bruker AXS, Karlsruhe, Germany) [77].

### 3.7. Statistics

All experiments described in this work were carried out in triplicates. The results obtained were subjected to statistical analysis using standard Microsoft Excel software, in which standard deviations and arithmetic means were determined. 

## 4. Conclusions

For the synthesis of PHA, three types of WFO obtained from the processing byproducts of Baltic sprats and Atlantic mackerel were studied for the first time. The compositions of the WFOs from the fatty acids varied; they included acids with chain lengths from C14 to C24 and had different degrees of saturation (from 0.52 to 0.65). While depending on these factors, all the types of waste supported the growth of the natural strain *C. necator* B-10646, which synthesized the PHA in the batch culture up to 67–72% (1.47–3.31 g/L). The bacterial biomass concentration varied, and it was comparable with the rates of bacterial growth on the WFOs from the heads of the smoked sprats and the heads and backbones of the fresh mackerel, respectively: 4.6. and 4.1. g/L. These results are comparable to those obtained under similar conditions in this strain when grown on glycerol, and twice as high as the waste from substandard fresh sprats. The synthesized PHAs, depending on the type of WFO, were represented by the P(3HB) homopolymer or the more technologically advanced P(3HB-*co*-3HV-*co*-3HHx) terpolymer, which had a reduced degree of crystallinity (71%). The results allow us to conclude that the studied waste-fish oils obtained from the smoked-sprat and fresh-mackerel waste are promising as accessible and renewable carbon substrates for PHA biosynthesis. 

## Figures and Tables

**Table 1:** Chemical composition of oils obtained from various byproducts (or WFOs) (% of absolutely dry matter).

Source of WFO	Total Lipids	Carbohydrates	Total Nitrogen	“Raw” Protein
Smoked-sprat heads	99.3	0.026	0.284	1.78
Raw sprats	86.2	0.024	0.309	1.93
Heads and backbones of fresh mackerel	89.6	0.025	0.503	3.14

**Table 2:** Fatty-acid composition of lipids derived from various WFOs.

Fatty Acids (FAs)	Content of Fatty Acids (% of Total Fatty Acids)		
	Fresh-Sprat Fat	Fat from Smoked-Sprat Heads	Fat from Heads and Backbones of Fresh Mackerel
14:0	3.41	3.51	2.82
*i*-14:0	0.08	0.13	0.25
*ai*-14:0	0.17	0.05	0.12
15:0	0.52	0.50	0.57
*i*-15:0	0.12	–	0.13
16:1ɷ7	2.32	0.32	2.74
16:0	18.80	28.04	26.21
16:1	0.20	–	0.08
*i*-16:0	–	0.27	0.32
*ai*-16:0	0.28	0.25	–
16:0-7-CH_3_	0.61	–	0.60
17:0	0.56	–	0.74
17:1	0.33	0.36	0.37
16:0-3,7,11,15-CH_3_	0.45	–	0.07
*i*-17:0	0.24	–	0.12
18:0	7.69	4.53	6.40
18:1ɷ9	17.74	25.33	32.70
18:2ɷ6	0.75	2.54	0.42
18:1	0.31	–	0.06
18:3ɷ3	2.48	4.34	0.77
19:0	–	–	0.14
*i*-18:0	0.12	–	–
20:0	0.46	0.33	0.32
20:1ɷ9	4.92	1.12	–
20:2	0.18	0.43	0.15
20:5ɷ3	9.98	8.74	4.28
20:4	0.45	–	0.86
20:3	0.08	–	–
20:6	0.33	–	–
22:0	0.18	0.54	0.38
*ai*-22:0	–	–	0.13
22:1	2.61	–	–
22:0-11-CH_3_	0.51	–	–
22:5	–	–	0.62
22:6ɷ3	22.28	16.73	16.94
24:1ɷ9	0.76	1.54	0.71
24:4	0.12	–	–
∑saturated FAs	34.20	38.15	39.32
∑unsaturated FAs	65.80	61.85	60.68
∑saturated FAs/ ∑unsaturated FAs	0.52	0.62	0.65
∑monounsaturated FAs	29.19	28.67	36.66
∑polyunsaturated FAs	36.65	32.78	24.04
∑long-chain FAs	42.86	29.43	24.39

– not detected.

**Table 3:** Kinetic and production parameters of the *C. necator* B-10646.

Source of WFO	Specific Growth Rate, h^−1^	PHA-Specific -Synthesis Rate, h^−1^	Biomass Productivity, g/L·h	PHA Productivity, g/L·h
Fresh sprat heads	0.10	0.15	0.03	0.02
Fresh mackerel heads and backbones	0.13	0.17	0.06	0.04
Smoked-sprat heads	0.14	0.19	0.06	0.05

**Table 4:** PHA production by *Cupriavidus necator* B-10646 and other bacterial strains using different WFOs as carbon sources.

Bacterial strain	Substrate	Conditions	X, g/L	PHA, %	PHA, g/L	Reference
*Cupriavidus necator* B-10646	Fresh sprat heads	0.5-liter flasks, 72 h	2.2	67	1.47	This study
	Fresh mackerel heads and backbones		4.1	69	2.83	
	Smoked-sprat heads		4.6	72	3.31	
*Pseudomonas oleovorans* strains	Hydrolyzed crude -ollock oil	0.5-liter flasks, 72 h	1.7–4.7	6–53	0.1–2.5	[54]
*Bacillus subtilis* KP17254	Solid fish-waste extract	1.0-liter flasks, 72 h	2.3	70	1.62	[55]
*C. necator* TISTR 1095	Tuna-condensate waste	0.5-liter flasks, 96 h	1.2–7.5	8.3–50.6	0.1–3.8	[56]
*Salinivibrio* sp. M318	Waste fish oil from basa fish	0.25-liter flasks, 48 h	10.0	51.7	5.2	[51]
	Waste fish oil from basa fish + glycerol	10-litr bioreactor	69.1	51.5	35.6	
*Ralstonia* sp. isolates	Crude fish oil	Flasks, 48 h	0.59–1.61	7.4–50.1	0.05–0.72	[52]
*Ralstonia* sp. M19		Flasks	3.93	61.95	2.43	
		10-liter bioreactor	5.32	51.23	2.73	
*C. necator* H16	WFO from basa fish	3-liter bioreactor, 48 h	114.8	72.5	83.2	[53]

**Table 5:** Compositions and properties of PHA synthesized by *C. necator* B-10646 on various WFO sources.

Source of WFO	Composition of PHA, mol.%			M_n_, kDa	M_w_, kDa	Đ	C_x_, %	T_melt_, °C	T_degr_, °C
	3HB	3HV	3HHx						
Fresh-sprat fat	100	–	–	209	711	3.4	78	168	278
Fat from heads and backbones of fresh mackerel	100	–	–	175	650	3.7	74	158	261
Fat from smoked sprat heads	97.1	1.6	0.3	134	752	5.6	71	160 168	284

## Data Availability

All data are available in the paper.
